# Programmed ROS/CO-releasing nanomedicine for synergetic chemodynamic-gas therapy of cancer

**DOI:** 10.1186/s12951-019-0507-x

**Published:** 2019-06-13

**Authors:** Bin Zhao, Penghe Zhao, Zhaokui Jin, Mingjian Fan, Jin Meng, Qianjun He

**Affiliations:** Guangdong Provincial Key Laboratory of Biomedical Measurements and Ultrasound Imaging, National-Regional Key Technology Engineering Laboratory for Medical Ultrasound, School of Biomedical Engineering, Health Science Center, Shenzhen University, No. 1066 Xueyuan Road, Nanshan District, Shenzhen, 518060 Guangdong China

**Keywords:** Gas therapy, Nanomedicine, Drug delivery, Controlled release, Combination therapy

## Abstract

**Background:**

To improve the outcome of cancer treatment, the combination of multiple therapy models has proved to be effective and promising. Gas therapy (GT) and chemodynamic therapy (CDT), mainly targeting the mitochondrion and nucleus, respectively, are two emerging strategy for anti-cancer. The development of novel nanomedicine for integrating these new therapy models is greatly significant and highly desired.

**Methods:**

A new nanomedicine is programmed by successive encapsulation of MnO_2_ nanoparticles and iron carbonyl (FeCO) into mesoporous silica nanoparticle. By decoding the nanomedicine, acidity in the lysosome drives MnO_2_ to generate ROS, ·OH among which further triggers the decomposition of FeCO into CO, realizing the effective combination of chemodynamic therapy with gas therapy for the first time.

**Results:**

Acidity in the TEM drives MnO_2_ to generate ROS, ∙OH among which further triggers the decomposition of FeCO into CO, realizing the effective combination of CDT and CDGT. The co-released ROS and CO do damage to DNA and mitochondria of various cancer cells, respectively. The mitochondrial damage can effectively cut off the ATP source required for DNA repair, causing a synergetic anti-cancer effect in vitro and in vivo.

**Conclusions:**

The combination of CDT and CDGT causing a synergetic anti-cancer effect in vitro and in vivo. The proposed therapy concept and nanomedicine designing strategy might open a new window for engineering high-performance anti-cancer nanomedicine.

**Electronic supplementary material:**

The online version of this article (10.1186/s12951-019-0507-x) contains supplementary material, which is available to authorized users.

## Background

CO as an endogenous gaseous signalling molecule plays an important role in regulation of physiological functions and treatment of inflammation-related diseases including cancer, ischemia–reperfusion injury, stroke, myocardial infarction, etc. [1–6]. It has proven that CO mainly target cellular mitochondria to modulate cellular energy level and then induce the apoptosis of cancer cells. It was also found that CO could sensitize chemotherapeutic drugs to kill cancer cells more effectively, enabling the combination of CO gas therapy with traditional therapy models to enhance cancer therapy efficacy [7]. Recently, utilizing the intratumoral chemical energy source to drive the generation of cytotoxic reactive oxygen species (ROS, mainly including hydroxyl radical ·OH and singlet oxygen ^1^O_2_) brings into a concept of chemodynamic therapy (CDT) [8–13]. We have also discovered that intratumoral overexpressed H_2_O_2_ can trigger the release of CO from metal carbonyl compounds (a kind of CO-releasing molecules, CORMs) through a Fenton-like reaction [6, 14]. Therefore we propose a concept of chemodynamic gas therapy (CDGT), which is defined as the intratumoral chemicals-driven release of gas for cancer therapy. We visualize that intratumoral chemicals could simultaneously drive CDT and CDGT for combination therapy.

**Scheme 1:**
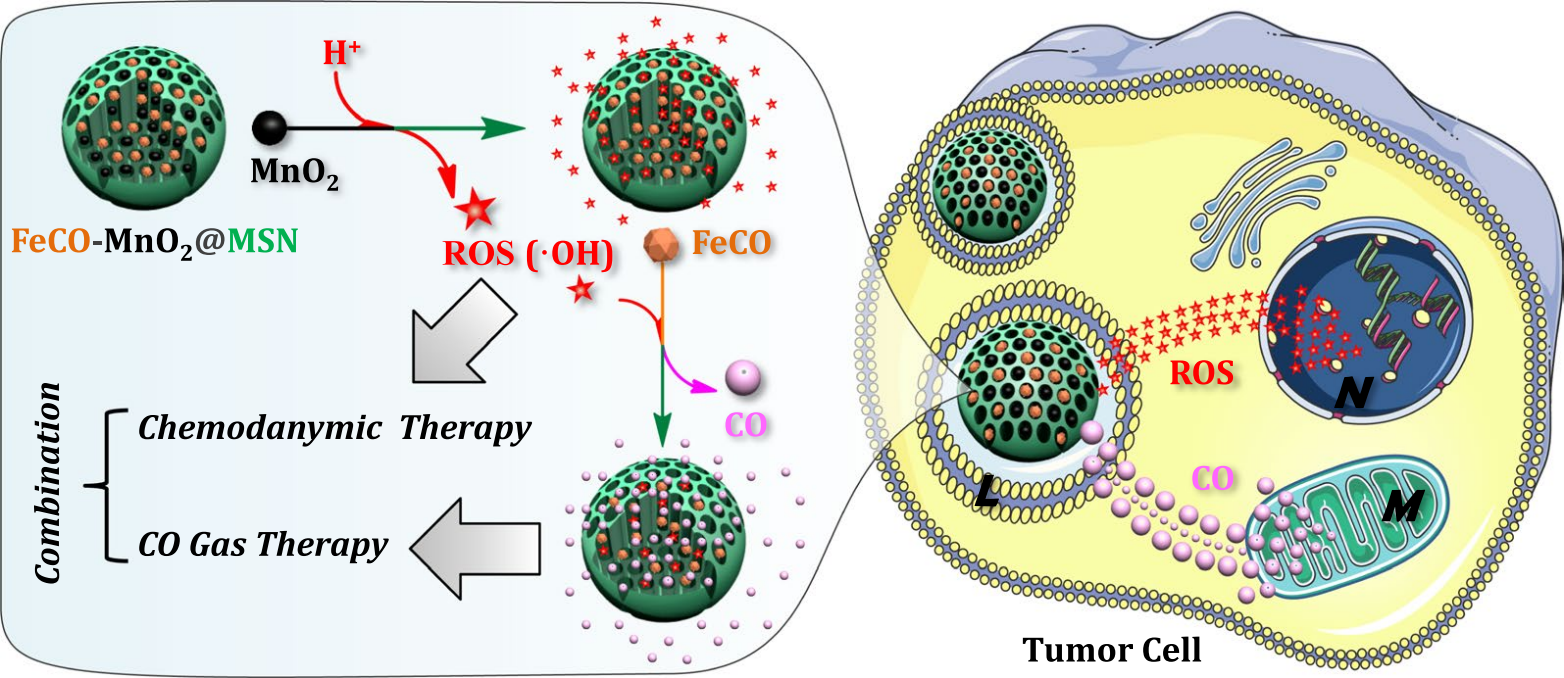
Schematic illustration of the mechanisms for acid-triggered sequential release of ROS and CO by the FeCO-MnO_2_@MSN nanomedicine for synergetic CDT and CDGT, which target the nucleus (N) and mitochondrion (M), respectively

In this work, an intelligent nanomedicine (FeCO-MnO_2_@MSN) was engineered and constructed by successively encapsulating MnO_2_ nanoparticles and iron carbonyl (FeCO) into mesoporous silica nanoparticle (MSN) to realize the intratumoral acid-triggered sequential release of ROS and CO for synergetic CDT and CDGT (Scheme 1), which was never reported before. Small-sized MnO_2_ nanoparticles were in situ loaded into the mesoporous channels of MSN during the synthesis of MSN, exhibiting high chemical reactivity to weak acidity in the TME. Amounts of ·OH were produced by a Fenton-like reaction for CDT, and subsequently in situ triggered the decomposition of co-loaded FeCO into CO for CDGT. Combined CDT and CDGT target cellular nucleus and mitochondrion to impair DNA and reduce energy metabolism, respectively, exhibiting unique effects of therapeutic synergy (Scheme 1). The combination of ROS and CO could generate a synergetic anticancer effect rather than a simple superposition of similar therapeutic effects. Such a synergetic strategy is quite novel. The design of the FeCO-MnO_2_@MSN nanomedicine based on this strategy was never reported previously.

## Results and discussion

**Fig. 1:**
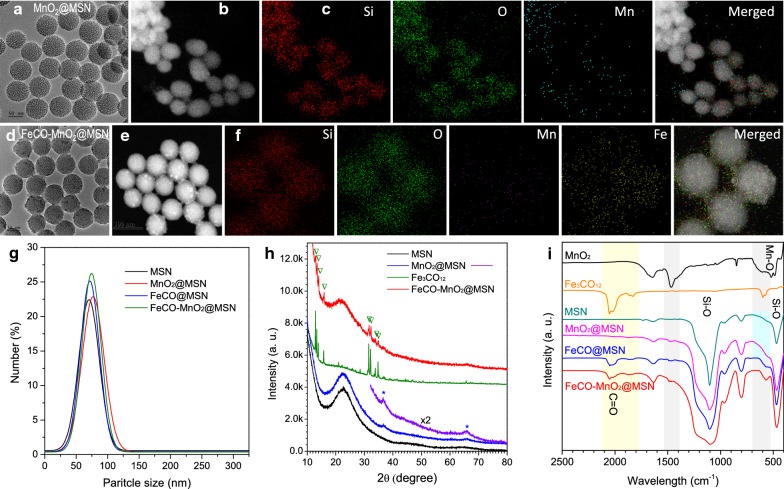
TEM (**a**, **d**), HADDF (**b**, **e**) and corresponding elementary mapping (**c**, **f**) images of MnO_2_@MSN (**a**–**c**) and FeCO-MnO_2_@MSN (**d**–**f**), DLS (**g**), XRD (**h**) and FTIR (**i**) patterns

To achieve the acid-derivated CDT, we designed a kind of highly sensitive MnO_2_@MSN nanomedicine for acid decomposition into ROS. In the process of MSN synthesis, the pore-forming template CTAC was also used as a reductant to in situ induce the confined growth of MnO_2_ nanoparticles within the mesopore channel of MSN by post-immersion in a potassium permanganate solution [15–17]. From the high-angle annular dark field (HADDF) and elementary mapping images of MnO_2_@MSN (Fig. 1b, c), it was found that a small size of MnO_2_ nanoparticles were plentifully (16.3 wt%) but stably dispersed within the mesoporous channels of MSN in favor of enhancing their reactivity and sensitivity to acid. Moreover, MnO_2_@MSN had high uniformity (about 70 nm in diameter, Fig. 1a, g), good mono-dispersity (Fig. 1g), high specific surface area (284.5 cm^2^/g, Additional file 1: Figure S1A) and open mesoporous channel (about 2.5 nm pore size and 0.55 cm^3^/g pore volume, Additional file 1: Figure S1B) in favor of further drug loading.

In order to avoid the leakage of drug from nanocarrier and to realize the responsive CO release, we chose Fe_3_(CO)_12_ (abbreviated as FeCO) as the solid state of CO prodrug because of its high hydrophobicity, relatively low toxicity among metal carbonyl-type donors of CO, high stability under physiological conditions and high sensitivity to ·OH. By a nano-casting method, FeCO was loaded into the synthesized MnO_2_@MSN (Fig. 1d–f), achieving a high drug loading capacity of 178 mg FeCO per gram silica (Additional file 1: Fig. S2) but still maintaining a good dispersivity in the PBS (Fig. 1g). The decreases in pore channel and surface area (171.5 cm^2^/g) of MnO_2_@MSN after FeCO loading (Additional file 1: Fig. S1) indicated that FeCO had been adsorbed into mesopores channels. XRD, FTIR and UV data further confirmed that both MnO_2_ and FeCO had indeed been encapsulated into MSN as indicated by their characteristic bands (* and ∇ symbols in Fig. 1h, yellow and gray zones in Fig. 1i, Additional file 1: Fig. S3).

**Fig. 2:**
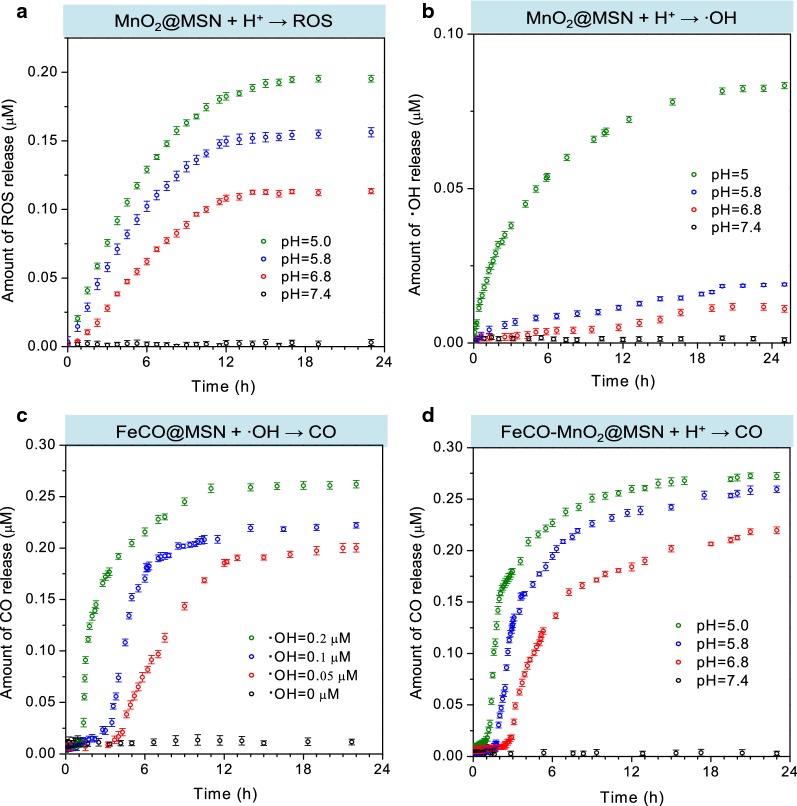
The sequential release behaviors: (i) acid-triggered release of ROS/·OH from MnO_2_@MSN (**a**, **b**), (ii) ·OH-triggered release of CO from FeCO@MSN (**c**), (iii) acid-triggered release of CO from FeCO-MnO_2_@MSN (**d**)

The ROS and CO release profiles of synthesized FeCO-MnO_2_@MSN nanomedicine were firstly evaluated in the PBSs with different pH values. We confirmed the sequential release process of ROS and CO through three substeps, (i) acid-triggered release of ROS/·OH from MnO_2_@MSN (Fig. 2a, b), (ii) ·OH-triggered release of CO from FeCO@MSN (Fig. 2c), (iii) acid-triggered release of CO from FeCO-MnO_2_@MSN (Fig. 2d). The concentrations of ROS, ·OH and CO were monitored in real time by UV/fluorescence spectrometry techniques using ABDA (9,10-anthracenediyl-bis(methylene) dimalonic acid) [18], coumarin [19] and Hb (hemoglobin) probes [4], respectively (Additional file 1: Figure S4–S9). From Fig. 2a, ROS could be released gradually from MnO_2_@MSN in an acid-responsive way. Higher acidity of PBS resulted in quicker release of ROS. Even at a slightly acidic condition with pH = 6.8, the ROS release was also distinct, while MnO_2_@MSN could keep stable in the pH = 7.4 PBS (Additional file 1: Figure S5), reflecting that MnO_2_@MSN had high sensitivity to acid owing to mesopores-confined small size of MnO_2_ (Fig. 2a–f). Meanwhile, the concentration of ·OH was also detected besides ROS. From Fig. 2b, an amount of ·OH (one of species in ROS) was also yielded concomitantly under the stimulation of acid. It should be attributed to the Fenton-like catalysis of MnO_2_ at the acidic condition for the generation of ·OH [11–13, 20–22]. Therefore, released ·OH could be used to further trigger the next reaction (Fig. 2c), while the residual ROS except ·OH could play the function of CDT.

The ·OH-triggered release of CO from FeCO@MSN was further checked. From Fig. 2c, it could be found that FeCO@MSN indeed could react with ·OH to decompose into CO, and higher concentration of ·OH could accelerate the CO release rate. Such a decomposition reaction was derived from the strong oxidizability of ·OH, which competed with carbonyl groups for coordination with Fe center. The ·OH-responsive CO release behavior of FeCO was similar to manganese carbonyl as we reported previously [6, 14]. By sequentially connecting these two reactions (Fig. 2b, c), we hypothesized that FeCO-MnO_2_@MSN would possibly release CO under the triggering of acid.

The acid-triggered release of CO from FeCO-MnO_2_@MSN was further checked. From Fig. 2d, it could be found that FeCO-MnO_2_@MSN could indeed release CO in an acid-responsive way as hypothesized. Higher acidity brought quicker release of CO from FeCO-MnO_2_@MSN, owing to above-confirmed two positive relationships of pH–·OH and ·OH–CO (Fig. 2b, c). The acid-triggered sequential release of ROS/·OH and CO provided the possibility for realizing the combination of CDT and CDGT. In addition, high responsiveness to acid and high stability in the pH = 7.4 PBS were helpful to avoid the CO leakage-caused risk of CO poisoning and also to contribute to the intratumoral release and accumulation of ROS/CO in favor of enhancing the efficacy of cancer therapy. In addition, the release curves could be by a double-Boltzmann model, suggesting that two kinds of physical collision among low concentrations of reactant particles played a predominant role (reaction-limited steps) over chemical reactions [4].

Intracellular uptake and ROS/CO release behaviors of FeCO-MnO_2_@MSN were further studied by microscopy monitoring technique. MSN was firstly labeled with RITC for red fluorescence, and then used to construct the nanomedicine FeCO-MnO_2_@MSN-RITC. HeLa cells were incubated with FeCO-MnO_2_@MSN-RITC (100 µg mL^−1^), then washed with PBS at the fixed time points, and stained with fluorescence probes and finally observed under confocal laser scanning microscopy (CLSM). DAPI, Lyso-Tracker Green, 2,7-dichlorodihydrofluorescein diacetate (DCFH-DA), 2-[6-(4,-hydroxy)phenoxy-3H-xanthen-3-on-9-yl]benzoic acid (HPF) [23] and COP-1 [4] were used to stain nuclei, lysosomes, ROS, ·OH and CO, respective.

**Fig. 3:**
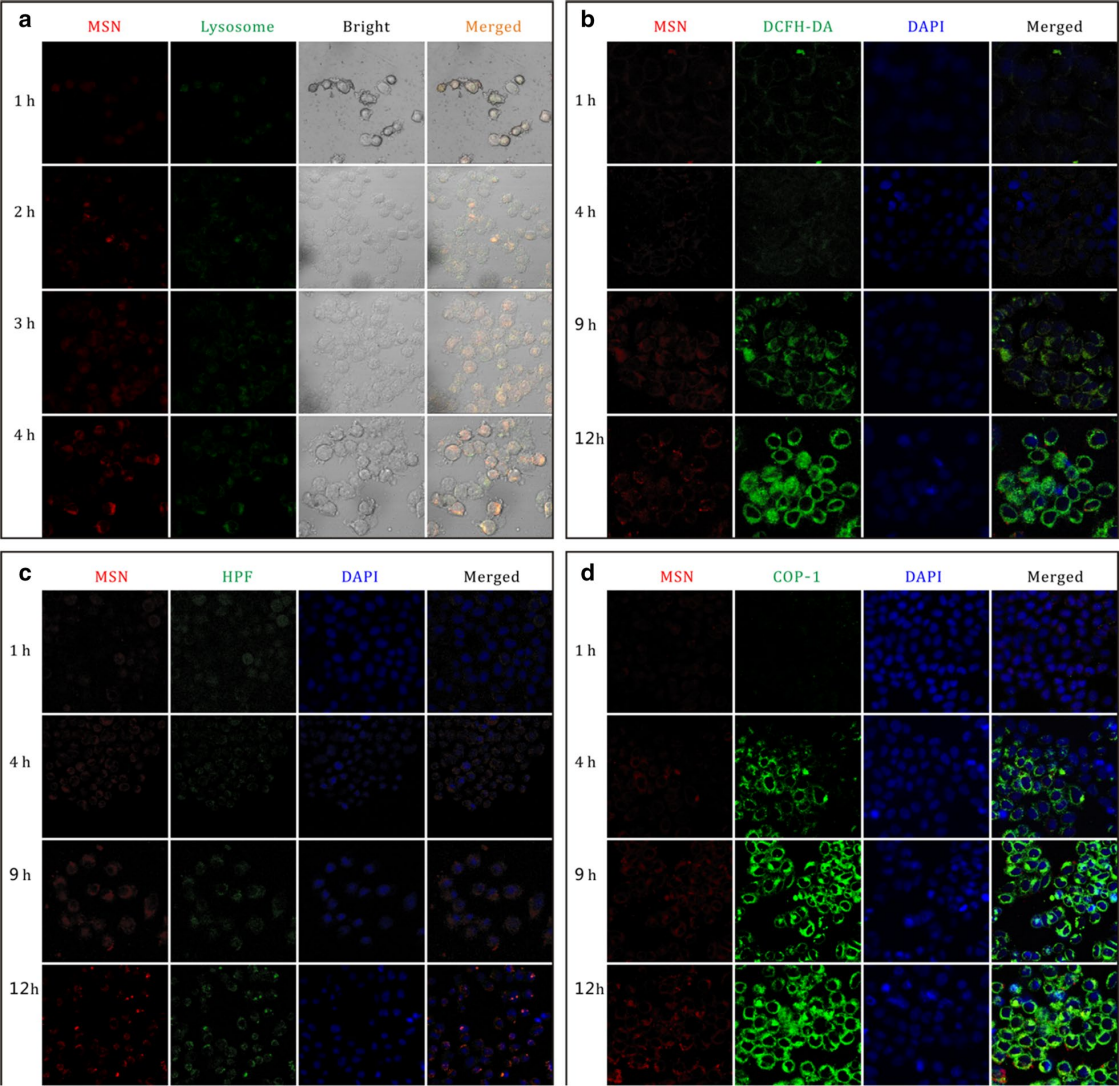
Confocal microscopy images of HeLa cells treated with FeCO-MnO_2_@MSN-RITC for different time periods and stained with varied fluorescence probes, Lyso-Tracker Green (**a**), DCFH-DA (**b**), HPF (**c**) and COP-1 (**d**), for detecting lysosomes, ROS, hydroxyl radicals and CO, respectively

From Fig. 3a, the red, green and yellow gradually increased with treatment time, indicating that FeCO-MnO_2_@MSN was gradually endocytosed into HeLa cells and translocated to the lysosomes. The highly acidic microenvironment in the lysosomes (pH ≈ 5) could provide a chemical driving force to trigger the sequential release of ROS/CO. From Fig. 3b, c, the gradual increase in the green suggested the intracellular generation and accumulation of ROS and ·OH, implying that the acidic microenvironment in the lysosomes could trigger the decomposition of endocytosed FeCO-MnO_2_@MSN into ROS/·OH. From Fig. 3d, it could also be found that CO was generated gradually in HeLa cells. The intracellular uptake of nanomedicine and the release of ROS/CO were well confirmed in accordance with the above-mentioned results in PBS (Fig. 2).

**Fig. 4:**
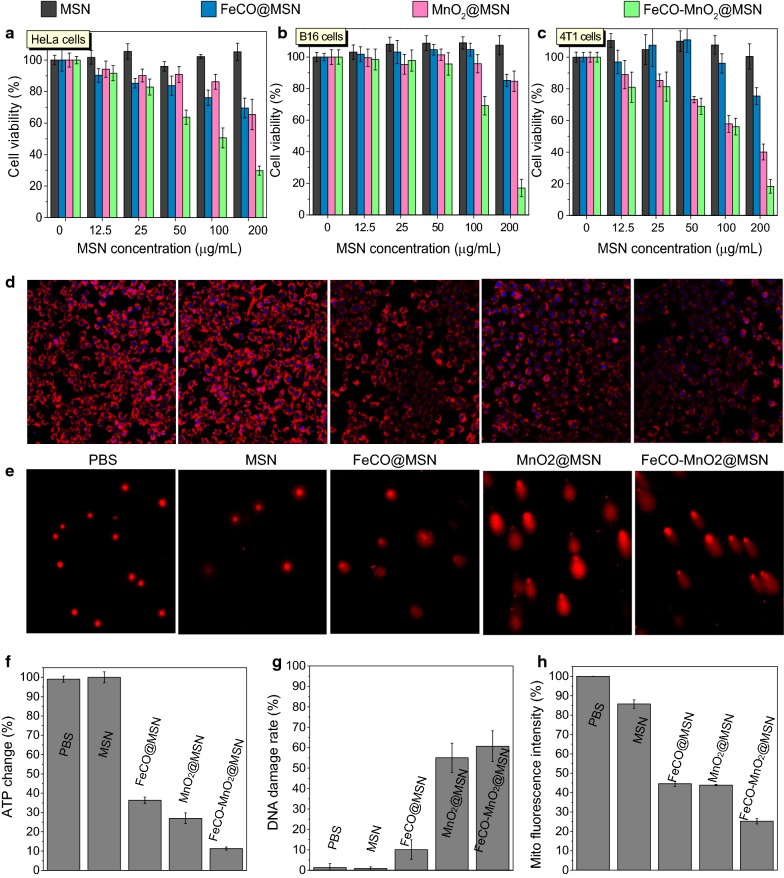
The cytotoxicity of MSN, FeCO@MSN, MnO_2_@MSN and FeCO-MnO_2_@MSN against HeLa, B16 and 4T1 cells 24 h after incubation (**a**–**c**), mitochondrial imaging with red fluorescence (**d**), Comet assay of DNA damage of 4T1 cells after drug treatment (**e**), the ATP levels in 4T1 cells (**f**). Figures **g** and **h** are statistic results of Figures **e** and **d**, respectively. Mean value and error bar are defined as mean and s.d., respectively

The cytotoxicity of FeCO-MnO_2_@MSN against various cancer cell (HeLa, B16 and 4T1 cell lines) was investigated using the CCK method to evaluate its therapy efficacies in vitro. From Fig. 4a–c, both MnO_2_@MSN and FeCO@MSN could cause the death of all these cancer cells to a certain extent generally, exhibiting that the anticancer outcomes of CDT and CDGT, respectively. By comparison, FeCO-MnO_2_@MSN showed much higher cytotoxicity, representing the synergetic anticancer effect of CDT and CDGT. It could be also found that the synergetic CDT/CDGT therapy was not dependent on the type of cancer cells, suggesting that the proposed strategy of CDT/CDGT combination therapy might be applicable to various cancers commonly. In addition, the cytotoxicity of FeCO-MnO_2_@MSN depended on drug concentration and treatment time duration. The prolongation of treatment time could result in higher therapy efficacy (Additional file 1: Figure S10), owing to the sustained ROS/CO release behaviors of FeCO-MnO_2_@MSN (Fig. 2). It could be found that almost all cancer cells had been killed 72 h after treatment with FeCO-MnO_2_@MSN at the concentration of 100–200 μg mL^−1^.

The mechanism for the CDT/CDGT synergetic therapy was further investigated from the aspects of cell energy metabolism [24] and DNA damage [25]. From Fig. 4f, the MSN carrier had no effect on the ATP level of 4T1 cells, while both MnO_2_@MSN and FeCO@MSN could clearly depress the ATP expression, suggesting that both ROS and CO could decrease the energy level of 4T1 cells. Cancer cells have higher energy level than normal cells owing to the Warburg effect [26, 27]. The damages to DNA and mitochondria by ROS and CO (Fig. 4d–h) blocked the energy metabolism of 4T1 cells (Fig. 4f), inducing cell apoptosis (Fig. 4c) [28–31]. By combination of CDT and CDGT, the FeCO-MnO_2_@MSN nanomedicine exhibited the synergistically enhanced inhibition to the energy of 4T1 cells (Fig. 4f) and reinforced damages to DNA and mitochondria (Fig. 4d, e, g, h), causing enhanced cytotoxicity to 4T1 cells (Fig. 4c). It could be found that the reduction in cellular energy level by CO suppressed the cellular self-repair of ROS-inducing DNA damage [32], promoting cell apoptosis and achieving the synergism of CDT/CDGT.

**Fig. 5:**
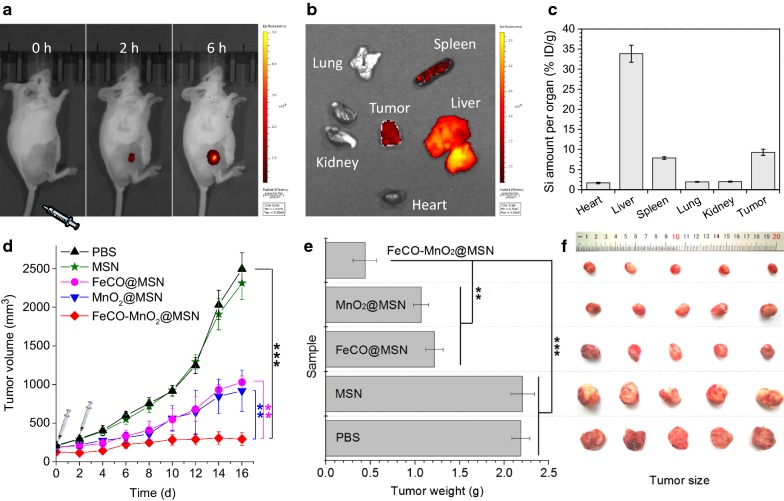
Fluorescence images of 4T1 tumor-loaded mice intravenously injected with/without the FeCO-MnO_2_@MSN nanomedicine (**a**), fluorescence images of extracted organs post injection (**b**), the biodistribution of the nanomedicine evaluated by the ICP technique (**c**), the change of 4T1 tumor volume during 16 day treatment (*n *= 5) (**d**), the weight (**e**) and size (**f**) of extracted tumors 16 days after treatment. Mean value and error bar are defined as mean and s.d., respectively. P values were calculated by the two-tailed Student’s t-test (***P < 0.005, **P < 0.05)

Encouraged by high therapy outcome in vitro, we tried to employ this nanomedicine for in vivo tumor therapy. In order to study the accumulation behavior of nanomedicine in the tumor site, the FeCO-MnO_2_@MSN nanomedicine was labeled with red fluorescence (FeCO-MnO_2_@MSN-RITC) for fluorescence imaging of treated mice. A 4T1 tumor-bearing mice model was firstly built by injecting 4T1 cells into BALB/c mice, and then the nanomedicine was injected into the 4T1 tumor-bearing mice via tail vein. After 2 h or 6 h post injection, the mice were imaged by the fluorescence imaging technique, and subsequently main organs were extracted for the ICP evaluation of nanomedicine biodistribution. From Fig. 5a, intensive fluorescence was observed in tumor, suggesting efficient intratumoral enrichment of nanomedicine. Ex vitro imaging results further suggested that injected nanomedicine mainly accumulated in liver, spleen and tumor (Fig. 5b). ICP results in Fig. 5c further confirmed the biodistribution of nanomedicine that large numbers of the nanomedicine could be retained in tumor besides liver and spleen. That should result from the EPR effect of tumor as the size of synthesized nanoparticles was only about 70 nm in diameter (Fig. 1a). Such a passive targeting behavior provided a good opportunity for tumor-targeted drug delivery and acid-triggered drug release.

Furthermore, the treatment experiments by intravenous injection of the nanomedicine were executed. The mice bearing 4T1 tumors were randomly divided into five groups (PBS control, MSN, MnO_2_@MSN, FeCO@MSN, and FeCO-MnO_2_@MSN) to compare the outcomes of CDT, CDGT, and combined CDT/CDGT. From Fig. 5d, MSN did not affect the tumor growth, while both MnO_2_@MSN and FeCO@MSN could stably suppress the tumor growth to a slight extent. By comparison with MnO_2_@MSN and FeCO@MSN, FeCO-MnO_2_@MSN could more effectively inhibit the tumor growth, suggesting the synergetic effect of CDT and CDGT in accordance with in vitro results (Fig. 4). By measuring the weights and sizes of extracted tumors 16 days after treatment, the therapeutic effects of MnO_2_@MSN, FeCO@MSN and FeCO-MnO_2_@MSN were further confirmed, and FeCO-MnO_2_@MSN exhibited the best treatment performance in the present conditions (Fig. 5e, f).

At the end of treatment, main organs and tumors were dissected from all groups and were then stained with hematoxylin and eosin (H&E). No obvious damage to normal tissues was visible, suggesting good biocompatibility of the carrier and nanomedicines (Additional file 1: Figure S11). In addition, body weights of mice showed no significant difference among all the groups during 16 d of the therapeutic period (Additional file 1: Figure S12). A high dose of FeCO-MnO_2_@MSN (67 or 134 mg/kg) was intravenously injected into health mice (*n *= 3), and no mouse was found died after 15 days, and then their blood liquid samples were collected. Compared with the control group, the injection of 67 mg/kg FeCO-MnO_2_@MSN (five folds higher than therapy dose) had not caused significant change in several routine hematological indicators (RBC, WBC, HGB, MCHC, LYM, MCV, HCT and RDW-SD) and some regarding the liver and renal functions (ALP, ALT, AST, CREA and BUN), indicating that FeCO-MnO_2_@MSN had no visible hematological toxicity, hepatotoxicity and nephrotoxicity with a high safe dose. These results indicated no severe side effects of CDT/CDGT combined therapy.

## Conclusion

In summary, we have successfully developed the intelligent nanomedicine FeCO-MnO_2_@MSN for acid-triggered sequential release of ROS and CO, realizing synergetic CDT and CDGT. The synthesized nanomedicine has exhibited good tumor targeting, and can utilize the acidic microenvironment in lysosome as a trigger source to responsively generate ROS and CO in tumor cells. CDT and CDGT target the nucleus and mitochondrion of tumor cells, respectively, which causes that the combination of CDT and CDGT has remarkably improved the outcomes of cancer therapy in vitro and in vivo. Such a synergetic strategy of targeting different organelles by combining gas therapy with other therapy models opens a new window for high efficacy of cancer therapy.

## Methods

### Material

Tetraethyl orthosilicate (TEOS), triethanolamine (TEAH), coumarin (C_9_H_6_O_2_, 99%), 9,10-anthracenediyl-bis(methylene) dimalonic acid (ABDA), sodium dithionite (SDT) and decahydronaphthalene (98%) were purchased from Sigma-Aldrich. Potassium permanganate (97%) was obtained from Shanghai Chemical Co. Dichloromethane (99.99%) was purchased from Aladdin Industrial Inc. Dodecacarbonyltriiron (Fe_3_(CO)_12_, 96%) was purchased from Macklin Industrial Inc. All chemicals were used as received without any further purification. Hemoglobin (Hb) of bovine red blood cells was purchased from MP Biomedicals. DAPI, Lyso-Tracker Green, 2,7-dichlorodihydrofluorescein diacetate (DCFH-DA) and 2-[6-(4,-hydroxy)phenoxy-3H-xanthen-3-on-9-yl]benzoic acid (HPF) were purchased from Beyotime Biotechnology.

### Synthesis of MSN nanoparticles

2 g of CTAC and 0.02 g of TEAH were dissolved into 20 mL water and stirred at room temperature for 30 min. Then heat the mixture to 80 °C and stabilize for 15 min. After that, 1.5 mL TEOS was gradually added into the above mixed solution. After 1 h, the product was collected by centrifugation (12,000 r min^−1^), and was washed by ethanol twice to remove free CTAC and other residual reactants.

### Synthesis of MnO_2_@MSN and FeCO-MnO_2_@MSN nanomedicines

The above as-synthesized MSN was dispersed into 10 mL of deionized water and kept at 40 °C in an oil bath with magnetic stirring. After 15 min, 10 mL of 10 mM KMnO_4_ solution was slowly dropped into the above MSN solution and stirred continuously for another 4 h. The product MnO_2_@MSN was collected by centrifugation, and then washed three times with ultrapure water.

A nano-casting method was used to construct the FeCO-MnO_2_@MSN nanomedicine. 5 mg FeCO was completely dissolved into 5 mL MeOH solution of MnO_2_@MSN (5 mg mL^−1^) to get a dark green solution. The mixture solution was vortexed for 10 min, then light-sealed and degassed under vacuum until the solution volume decreased to about 500 μL. FeCO-MnO_2_@MSN nanoparticle was collected by centrifugation and then 5 mL of oxygen-free ultrapure water was immediately added and degassed under vacuum until no bubbles were generated to remove MeOH. The obtained FeCO-MnO_2_@MSN was washed with water several times.

### Characterization

TEM images of nanoparticles were collected on a JEM-2100F transmission electron microscope with an acceleration voltage of 200 kV. BET measurements were performed at 77 K using a Micromeritics Tristar 3020 Analyzer (USA). Confocal luminescence images were obtained using a Leica TCS SP8 confocal laser scanning microscope. Hydrodynamic size was measured on a Malvern Zetasizer Nano ZS90 equipped with a solid He–Ne laser (λ = 633 nm). The crystal phase structures of MSN, MnO_2_@MSN, Fe_3_CO_12_ and FeCO-MnO_2_@MSN were characterized by powder X-ray diffraction (XRD, M21X). The diffractometer (CuKα, λ = 1.54056 Å) was operated at 40 kV and 200 mA. XRD patterns were collected in a scanning range of 10°–80° at room temperature. FT-IR spectra were collected on a Thermo-Nicolet Nexus 670 ATR-IR spectrometer. UV spectra were recorded on a Genesys 10S UV–Vis spectrophotometer (Thermo Sci.) at room temperature. Fluorescence Spectrophotometer (Thermo Sci.) was used for fluorescence detection. The UV method was used to measure the FeCO loading capacity of MnO_2_@MSN. Different concentrations of the methanol solutions of FeCO were first detected by a UV spectrophotometer to obtain a standard curve as shown in Additional file 1: Figure S2. The 600 nm absorbances of the above supernatant solutions before and after FeCO loading were measured and used to calculate the FeCO loading capacity using the standard curve according to the Beer-Lambert’s law, as shown in Additional file 1: Figure S2. The FeCO loading capacity was calculated to be 178 mg FeCO per gram silica.

### Acid-triggered ROS/CO release measurement

ABDA was used to detect ROS in the simulated solutions. In a typical experiment, the solution of MnO_2_@MSN (12.8 μg) was added to the PBS solution of ABDA (0.25 μM, 3 mL) with different pH values and placed in a cuvette. The change in ABDA absorbance at 400 nm was recorded as a function of time via UV–vis spectrometry. The ROS level was measured according to the standard curve of ABDA (Additional file 1: Figure S4).

Coumarin can readily react with hydroxyl radicals to produce highly fluorescent product, 7-hydroxycoumarin. In a typical process, MnO_2_@MSN (15 μg) was added to the PBS solutions of Coumarin (0.5 μM, 3 mL) with different pH values (pH = 5.0, 5.8, 6.8, 7.4). After a certain period of time, the reaction solution was filtered to measure the increase in the photoluminescence intensity at 445 nm (excited at 332 nm).

Hemoglobin (Hb) was used to detect the release of CO in PBS. As shown in the protocol (hemoglobin reaction diagram). First, SDT was added to reduce Hb, and then, Hb was completely dissolved in the Ultra-pure water of different concentrations of hydroxyl radicals (0 μM, 0.05 μM, 0.1 μM, 0.2 μM) or the PBS solutions of different pH values (pH = 7.4, 6.8, 5.8, 5.0). FeCO@MSN (14.8 μg) and FeCO-MnO_2_@MSN (15 μg) were then added to the above ·OH and acidic solutions, respectively. All 3 mL of the reaction solution was immediately sealed in a 4 mL UV quartz cuvette. The UV absorption spectra of the solution (350–600 nm) were collected on a Genesys 10S UV–Vis spectrophotometer (Thermo Sci.) at fixed time points. Two adsorption bands at 410 nm and 430 nm, which were attributed to HbCO and Hb, respectively, were used to quantify the conversion of Hb to HbCO. The Beer–Lambert law was used to calculate the concentration of released CO which was coordinated with Hb, as indicated by following functions.

$$C_{CO} = \frac{{528.6 \times I_{{410\;{\text{nm}}}} - 304 \times I_{{430\;{\text{nm}}}} }}{{216.5 \times I_{{410\;{\text{nm}}}} + 442.4 \times I_{{430\;{\text{nm}}}} }}C_{Hb}$$

wherein, C_CO_ and C_Hb_ express the released CO concentration and the initial Hb concentration (4.2 μM), respectively [18].

### Cell culture

HeLa, B16 and 4T1 cell lines were cultured in the regular growth medium consisted of DMEM (minimum essential medium) supplemented with 10% FBS (fetal bovine serum) at 37 °C and 5% CO_2_ incubator.

### Measurement of cellular and lysosomes uptakes of nanomedicine

HeLa cells were seeded at a density of 1 × 10^5^ cells per well in 6-well plates containing slides and incubated at 37 °C. After 48 h, the medium containing FeCO-MnO_2_@MSN-RITC (100 μg mL^−1^) was subjected to an uptake study for 1, 2, 3, 4 h. The supernatant was removed, and the cells were washed three times. The lysosomal/nucleus probes Lyso-Tracker Green and DAPI were added at the final concentration of 2 μmol L^−1^ and 1 μg mL^−1^, respectively. And then cells were observed on a confocal microscope.

### Measurement of intracellular ROS, ·OH and CO levels

Intracellular ROS in HeLa cells was detected with a ROS indicator DCFH-DA. 100 μg mL^−1^ FeCO-MnO_2_@MSN was incubated with HeLa cells for various time periods. DCFH-DA and DAPI were added at the final concentration of 20 μmol L^−1^ and 1 μg mL^−1^, respectively. Then the intracellular fluorescence was observed by confocal laser scanning microscopy.

Intracellular ·OH in HeLa cells was detected with a ·OH indicator HPF. FeCO-MnO_2_@MSN (100 μg mL^−1^) was incubated with HeLa cells for various time periods. HPF and DAPI were added at the final concentration of 2 μmol L^−1^ and 1 μg mL^−1^, respectively. Then the intracellular fluorescence was observed by confocal laser scanning microscopy.

Intracellular CO in HeLa cells was detected with a CO probe COP-1. FeCO-MnO_2_@MSN (100 μg mL^−1^) was incubated with HeLa cells for various time periods. COP-1 and DAPI was added, with the final concentration of 20 μmol L^−1^ and 1 μg mL^−1^, respectively. Then the intracellular fluorescence was observed by confocal laser scanning microscopy.

### Intracellular ATP level measurement

An ATP kit was used to measure the intracellular ATP level. 4T1 cells were seeded at a density of 1 × 10^5^ cells per well in 6-well plates for 24 h at 37 °C. The cells were treated with FeCO-MnO_2_@MSN at the concentration of 100 μg mL^−1^. After 24 h, the cells were lysed and centrifuged. Thus, intracellular ATP can be obtained from the supernatant collected after lysis centrifugation. The firefly luciferase and fluorescein mixture was used as a probe for detecting ATP. Firefly luciferase catalyzes the fluorescein to produce fluorescence, requiring ATP to provide energy. When there is an excess of luciferase and fluorescein, the ATP concentration is linearly related to the fluorescence intensity. The ATP level was calculated according to the change of fluorescence intensity.

### Intracellular mitochondrial damage level measurement

4T1 cells (10^5^ cells per dish) were seeded in a cover glass dish (35 mm × 10 mm). The cells were treated with FeCO-MnO_2_@MSN at a concentration of 100 μg mL^−1^. MitoTracker Red CMXRos and Hoechst solutions were added to stain the mitochondria and nuclei of 4T1 cells, respectively. Finally, the cells were gently washed twice, 1 mL of HEPES solution was added, and the cells were observed under a confocal laser scanning microscope.

### Cell cytotoxicity assay by CCK-8 protocol

In vitro cytotoxicity was assessed by the CCK-8 assay. Tumor cells were seeded in 96-well plates at a density of 104 cells per well and cultured in 5% CO_2_ at 37 °C for 24 h. Then, the culture medium was discarded, and the cells were then treated with 100 μL DMEM solution of FeCO-MnO_2_@MSN at final concentrations of 12.5–200 μg mL^−1^. At the end of the incubation (24 h, 48 h, 72 h), the medium was removed, and 100 μL of fresh medium and 10 μL of CCK-8 solution were added in turn and incubated for another 2 h, and the absorbance at 450 nm was monitored using a microplate reader. The cytotoxicity was expressed as the percentage of cell viability compared to the blank control group.

### Tumor modeling and in vivo anti-cancer effect

The Administrative Committee on Animal Research in Shenzhen University approved the protocols for all animal experiments. 4T1 cells (2 × 10^6^ cells/site) were subcutaneously injected into the thigh root of 4–5 week Balb/c mice (~ 20 g, purchased from Guangdong Medical Laboratory Animal Center). 4T1 tumor-bearing Balb/c mice were randomly divided in five groups to be injected with PBS (n = 5), MSN, MnO_2_@MSN, FeCO@MSN and FeCO-MnO_2_@MSN (10 mg MSN per kg mice), respectively, through the tail vein when the tumor reached about 100 mm^3^. After treatment for 16 days, heart, liver, spleen, lung and kidney were harvested for H&E staining assay. All the animals in this study received humane care in compliance with the institution’s guidelines for the maintenance and use of laboratory animals in research. Animal procedures involving animals in this study were in accordance with ethical standards and approved by the Institutional Animal Care and Use Committee of Shenzhen University.

### Tumor targeting of nanomedicine

FeCO-MnO_2_@MSN-RITC nanomedicine was injected into 4T1 tumor-bearing Balb/c mice through the tail vein. The biodistribution of nanomedicine was observed by fluorescence imaging after 6 h. Then, heart, liver, spleen, lung and kidney were harvested for ICP quantitative detection of nanomedicine accumulation (n = 3).

### The liver/kidney function and hemotoxicity analyses

BALB/c mice were randomly divided into 3 groups (*n *= 3) to be injected with 100 μL PBS (as control), 100 μL 67 mg kg^−1^ FeCO-MnO_2_@MSN, 100 μL 134 mg kg^−1^ FeCO-MnO_2_@MSN. After 15 days, the blood was collected and detected by a biochemical analyzer (iMagic-M7) and a blood cell analyzer (BC-31s, Mindray).

## Additional file

**Additional file 1: Figure S1.** Nitrogen adsorption–desorption isotherms. **Figure S2.** The standard curve of FeCO. **Figure S3.** UV spectra of the carrier, drug and nanomedicine. **Figure S4.** The ADBA standard curve for calculation of ROS concentration. **Figure S5.** The monitoring of ROS release in different pH PBSs using ADBA as probe by the UV method. **Figure S6.** The coumarin standard curve for calculation of ∙OH concentration. **Figure S7.** The monitoring of ∙OH release in different pH PBSs using coumarin as probe by the UV method. **Figure S8.** The monitoring of CO release in different ∙OH concentrations using Hb as probe by the UV method. **Figure S9.** The monitoring of CO release in different acidic solutions using Hb as probe by the UV method. **Figure S10.** Cytotoxicities of different concentrations of nanomedicines to various cancer cells. **Figure S11.** Histological examination of main organs (heart, liver, spleen, lung and kidney) from treated mice by the HE staining method. **Figure S12.** The weight change of 4T1 tumor-bearing mice during treatment. **Figure S13.** Blood biochemical analyses. **Figure S14.** The evaluation of standard haematology markers.

## Data Availability

All data generated or analyzed during this study are included in the article and additional file.
