# Supplementary material for: Programmed ROS/CO-releasing nanomedicine for synergetic chemodynamic-gas therapy of cancer
Source: J Nanobiotechnology. 2019 Jun 13;17:75. doi: 10.1186/s12951-019-0507-x (PMC6567615; doi:10.1186/s12951-019-0507-x)
Supplement: Supplementary file 1 — Additional file 1: Figure S1. Nitrogen adsorption–desorption isotherms. Figure S2. The standard curve of FeCO. Figure S3. UV spectra of the carrier, drug and nanomedicine. Figure S4. The ADBA standard curve for calculation of ROS concentration. Figure S5. The monitoring of ROS release in different pH PBSs using ADBA as probe by the UV method. Figure S6. The coumarin standard curve for calculation of ∙OH concentration. Figure S7. The monitoring of ∙OH release in different pH PBSs using coumarin as probe by the UV method. Figure S8. The monitoring of CO release in different ∙OH concentrations using Hb as probe by the UV method. Figure S9. The monitoring of CO release in different acidic solutions using Hb as probe by the UV method. Figure S10. Cytotoxicities of different concentrations of nanomedicines to various cancer cells. Figure S11. Histological examination of main organs (heart, liver, spleen, lung and kidney) from treated mice by the HE staining method. Figure S12. The weight change of 4T1 tumor-bearing mice during treatment. Figure S13. Blood biochemical analyses. Figure S14. The evaluation of standard haematology markers. [file 12951_2019_507_MOESM1_ESM.docx]

**Additional Information**

**Programmed ROS/CO-Releasing Nanomedicine for Synergetic Chemodynamic-Gas Therapy of Cancer**

Bin Zhao, Penghe Zhao, Zhaokui Jin, Mingjian Fan, Jin Meng and Qianjun He*

**Results and Discussion**

**Figure S1.** Nitrogen adsorption–desorption isotherms (A) and pore size distribution curves (B) of MSN before and after encapsulation with MnO_2_ and FeCO.

**Figure S2.** The standard curve of FeCO (B), plotted according to the UV adsorption of FeCO solutions with different concentrations (A).

**Figure S3.** UV spectra of the carrier, drug and nanomedicine.

**Figure S4.** The ADBA standard curve for calculation of ROS concentration (B), plotted according to the UV adsorption of ADBA solutions with different concentrations (A).

**Figure S5.** The monitoring of ROS release in different pH PBSs using ADBA as probe by the UV method.

**Figure S6.** The coumarin standard curve for calculation of ∙OH concentration (B), plotted according to the UV adsorption of coumarin solutions with different concentrations (A).

**Figure S7.** The monitoring of ∙OH release in different pH PBSs using coumarin as probe by the UV method.

**Figure S8.** The monitoring of CO release in different ∙OH concentrations using Hb as probe by the UV method.

**Figure S9.** The monitoring of CO release in different acidic solutions using Hb as probe by the UV method.

**Figure S10.** Cytotoxicities of different concentrations of nanomedicines to various cancer cells (HeLa, 4T1 and B16 cells) after treatment for 48 h and 72 h.

**Figure S11.** Histological examination of main organs (heart, liver, spleen, lung and kidney) from treated mice by the HE staining method.

**Figure S12.** The weight change of 4T1 tumor-bearing mice during treatment.

**Figure S13.** Blood biochemical analyses including liver functions (A‒C) and kidney functions (D,E). ALP, alkaline phosphatase; ALT, alanine transaminase; AST, aspartate transaminase; CREA, creatinine; BUN, blood urea nitrogen (*n* =3). Mean value and error bar are defined as mean and s.d., respectively. *P* values were calculated by the two-tailed Student’s *t*-test (**P*<0.05, ns: no significant difference) by comparison with the control. When the injection dose was as high as 134 mg/kg (ten folds higher than therapy dose), BUN value was significantly higher than the control, suggesting clear influence on kidney.

**Figure S14.** The evaluation of standard haematology markers including RBC (A), WBC (B), HGB (C ), MCHC (D ), MPV (E), MCV (F), LYM (G), HCT (H) and RDW-CV (I). RBC, red blood cell content; WBC, white blood cell content; HGB, haemoglobin concentration; MCHC, mean corpuscular haemoglobin concentration; MPV, mean platelet volume; MCV, mean corpuscular volume; LYM, lymphocytes percentage; HCT, haematocrit; RDW-CV, red blood cell volume distribution width (*n* = 3). Mean value and error bar are defined as mean and s.d., respectively.
